# Idiopathic Generalized Epilepsy and Hypokalemic Periodic Paralysis in a Family of South Indian Descent

**DOI:** 10.1155/2015/906049

**Published:** 2015-03-26

**Authors:** Muthiah Subramanian, N. Senthil, S. Sujatha

**Affiliations:** Department of General Medicine, Sri Ramachandra University, No. 1 Ramachandra Nagar, Porur, Chennai 600116, India

## Abstract

Inherited channelopathies are a heterogeneous group of disorders resulting from dysfunction of ion channels in cellular membranes. They may manifest as diseases affecting skeletal muscle contraction, the conduction system of the heart, nervous system function, and vision syndromes. We describe a family of South Indian descent with hypokalemic periodic paralysis in which four members also have idiopathic generalized epilepsy. Hypokalemic periodic paralysis is a genetically heterogeneous channelopathy that has been linked to mutations in genes encoding three ion channels CACNIAS, SCN4A, and KCNJ2 predominantly. Although data on specific gene in idiopathic generalized epilepsy is relatively scarce, mutations of voltage gated sodium channel subunit genes (CACNB4) and nonsense mutations in voltage gated calcium channels (CACNA1A) have been linked to idiopathic generalized epilepsy in two families. We speculate that gene mutations altering the ability of the beta subunit to interact with the alpha subunit of the CaV1.1 channel and mutations in the pore-forming potassium channel subunit may be possible explanations for the combined manifestation of both diseases. Functional analysis of voltage gated calcium channel and other ion channels mutations may provide additional support and insight for the causal role of these mutations. The understanding of mutations in ion-channel genes will lead to improved diagnosis and treatment of such inherited channelopathies.

## 1. Introduction

Hypokalemic periodic paralysis is a rare inherited autosomal dominant disorder with an overall incidence of 0.4–1 cases per 100,000 [1]. Although periodic paralysis is seldom life threatening, it is characterized by episodic flaccid weakness with concomitant fall in blood potassium levels. Mutations in the sodium channel protein alpha subunit gene SCN4A on chromosome 17q23.3 and L-type calcium channel alpha-1S subunit gene CACNA1S on chromosome 1q32.1 are the most common inherited form of hypokalemic paralysis [2]. There is increasing evidence that inherited ion-channel dysfunction contributes to the molecular basis of epilepsy. Idiopathic generalized epilepsy (IGE) constitutes roughly one-third of all epilepsies and is “forms of generalized epilepsies in which all seizures are initially generalized with an EEG expression that is a bilateral, synchronous discharge” [3]. The IGEs comprise several subsyndromes including typical absences, myoclonic jerks, and generalized tonic clonic seizures. Both familial and de novo mutations in neuronal voltage-gated channel subunit genes have been identified in idiopathic generalized epilepsy [4]. We describe a South Indian family with hypokalemic periodic paralysis in which four members also have idiopathic generalized epilepsy and discuss the possibility that dysfunction of voltage gated calcium channels may be associated with both disorders.

## 2. Case Report

**Figure 1:**
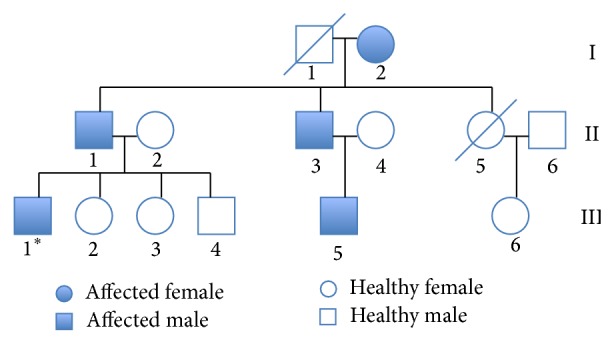
The family genealogical tree. Filled symbols indicate patients with hypokalemic periodic paralysis and idiopathic generalized epilepsy. ^∗^This patient has only been affected with hypokalemic periodic paralysis.

The family described in this case report is of South Indian descent. The disease was documented over three generations and the relevant part of the pedigree is described in Figure 1. The diagnosis of hypokalemic periodic paralysis was based on appropriate clinical history, laboratory investigations, and/or electromyography as described in Table 1. EEG recordings were carried out on four of the individuals. Clinical history, witness account, and EEG recordings were used to confirm the diagnosis of epilepsy.

### 2.1. Case III 5

A 26-year-old male had recurrent attacks of proximal and distal muscle weakness for the last 5 years. Since the age of 21 he has multiple episodes of weakness frequently following strenuous exercise. He came to the emergency room on December 15, 2012, with sudden onset paralysis following participating in a cricket match. There was bilateral weakness of both proximal and distal muscles. He had no neck or facial muscle weakness and had no difficulty with swallowing. He denied any history of recent diarrhea, chest pain, or shortness of breath. He had been suffering from seizures since the age of 19 and had been on regular antiepileptic drugs. The last witnessed seizure was eleven months ago after which his local physician had increased the dose of his AEDs. He did not have any other significant past medical history and denies use of alcohol and drugs.

On physical examination, he was moderately built and otherwise normal in overall appearance. There was no jugular venous distension, goiter, or lymphadenopathy. There was hypotonia, flaccid paralysis of all extremities, and depressed deep tendon jerks. Cardiovascular examination revealed a regular pulse with no murmurs. Other system examinations were unremarkable.

Routine hemogram, biochemistry, and liver enzymes were within normal limits except for a serum potassium level of 1.9 mmol/L. In search of an etiology of hypokalemia, urine sodium and potassium were measured and found to be normal. Serum renin and aldosterone and thyroid function tests were also measured to rule out adrenal and coexistent thyrotoxic periodic paralysis, respectively. A routine electrocardiogram revealed the presence of a prolonged PR interval with U waves. EMG revealed total electric silence. Following intravenous potassium supplementation he made a complete recovery within 48 hours with dramatic improvement of muscle power and deep tendon reflexes.

On the fifth day of admission, he developed a convulsion with clonic movement of both arms and legs with tonic spasms of the neck muscles. The patient was afebrile and had no neck stiffness. A complete haemogram and biochemistry revealed a normal serum potassium, sodium, and blood sugar. A lumbar puncture showed normal findings. A postictal electroencephalogram study revealed synchronous generalized polyspike-and-wave discharges with normal background. Fosphenytoin and valproic acid were used to control the epileptiform discharges.

### 2.2. Case I 2

The 62-year-old grandmother of the proband (Case III 5) had similar complaints of weakness since the age of 32 years. She recalls presenting to a tertiary care hospital at 33 years old and being told that her serum potassium was low. The doctor recommended further evaluations such as an EMG study; however she did not follow up for additional testing and was lost to follow-up. She has been on treatment with various antiepileptic drugs for the last 31 years. Her last witnessed seizure was 4 years ago at which time she had tonic clonic contractions of her extremities with eyelid flickering and lip smacking. The patient was treated with carbamazepine and had no further seizures. An interictal EEG was not done. An MRI scan of the brain was normal. Serum electrolytes, calcium, and magnesium were normal.

### 2.3. Case II 1

The 32-year-old father of the proband (III 5) began to have epileptic seizures since the age of 16. The clinical features suggest that the patient suffered from partial complex seizures with secondary generalization. The episodes last approximately 2 minutes. The seizures have been poorly controlled with a combination of phenytoin and sodium valproate. An interictal EEG at the time of diagnosis reveals rhythmical slow-wave activity over the bilateral hemispheres becoming spike and slow-wave complexes. A CT scan of the brain was normal.

Following a binge of alcohol at the age of 25 he was admitted with sudden weakness of all four limbs with difficulty in respiration. He had 5 similar attacks within the previous 2 years; however the severity of the weakness had never warranted admission. His serum potassium during the attack was 2.1 mEq/L. Urine spot potassium and thyroid function tests were normal. Electromyography performed in the lower limbs showed decreased compound motor action potentials. He continues to have these episodes a few times a year.

### 2.4. Case II 3

The 48-year-old male had hypokalemic periodic paralysis and epilepsy diagnosed at 20 and 18 years, respectively. His episodes of hypokalemic periodic paralysis were more frequent as a teenager and occurred mainly at night. He had a low ictal serum potassium (2.2 mEq/L) and EMG demonstrating complete electrical silence. Now the attacks occur mainly after stress or strenuous physical exercise. He complains of weakness attacks once or twice a month.

At the age of 18 he was admitted for evaluation of multiple syncopal attacks that lasted for a few minutes followed by postictal confusion and headache. During his admission he had a convulsion with tonic-clonic movements of both arms and jaw muscles. His EEG revealed bilateral bursts of spike-wave activity that were increased during hyperventilation. He was diagnosed with idiopathic generalized epilepsy. His serum electrolytes, blood sugar, and blood count were within normal limits. The epileptic seizures are well controlled with sodium valproate.

### 2.5. Case III 1

The 11-year-old boy has had complaints of intermittent weakness in his legs since the age of 4 years. His legs would buckle under him especially during the summer months and after exercises. He attends a school for children with mild to moderate learning difficulties. At the age of 7 years he was admitted following an attack of sudden paralysis that appeared in the early morning when he was awakened. The weakness was initially in the lower limbs and lasted several hours. The serum potassium at the time of weakness was 1.9 mEq/L. An EMG showing compound motor unit action potentials of very low amplitudes supported a diagnosis of hypokalemic periodic paralysis. The boy continues to have episodes of weakness once or twice a week. However, the patient has not experienced any seizures.

## 3. Discussion

We have reported a family with hypokalemic periodic paralysis in which four individuals have idiopathic generalized epilepsy. The age of onset of hypokalemic periodic paralysis was 20 to 32 years except in the proband III I in whom the attacks started at the age of 4 years. The periodic paralysis was precipitated by prolonged exercise, carbohydrate meals, and alcohol and during exposure to stress. The frequency of episodes varied ranging from 1-2 times a week to once a year. The paralytic episodes never lasted more than 24 hours in each case. The ictal serum potassium was low (1.9 mEq/L–2.3 mEq/L) in all four individuals in whom evaluation was undertaken. The most helpful investigation was the EMG that showed complete electrical silence (II 3, III 5) in two patients and decreased compound motor action potentials in the others (II 1, III 1). Thyroid function tests, renin, aldosterone, and other serum electrolytes were normal in all individuals. A combination of oral and intravenous correction of serum potassium showed dramatic improvement in muscle power and deep tendon reflexes. The age of onset of epileptic seizures was comparatively earlier than the onset of periodic paralysis in all affected family members. The clinical presentation was similar in the 4 effected patients with tonic- clonic convulsions of arms and legs lasting for a few minutes followed by postictal confusion. There were bisynchronous, symmetric, and generalized spike-wave complexes in the postictal EEG of cases II 1, II 3, and III 5. The strong family history, clinical presentation, and EEG findings were important clues that led to a diagnosis of generalized idiopathic epilepsy in these patients. The seizures were well controlled with phenytoin and sodium valproate except in proband II 1 in whom seizures persist despite multiple antiepileptic drugs.

The mean age of onset of paralytic attacks in patients with inherited hypokalemic periodic paralysis is 5 ± 5 years [5]. In the reported family the clinical onset of periodic paralysis was not until early adulthood. In some families with inherited channelopathies such as erythromelalgia, clinical onset is delayed until adulthood [6, 7]. The late onset presentation of inherited channelopathies may reflect the presence of compensatory mechanisms, early in life, which suppress clinical manifestations. Compensatory expression of other channels which act to maintain excitability at close-to-normal levels or altered interactions between subunits of ion channels may be considered as possible explanations [8]. Further evaluation is needed to elucidate the possible molecular protective mechanisms in this family.

Hypokalemic periodic paralysis is an autosomal dominant, genetically heterogeneous channelopathy. Family history is often underestimated due to nonpenetrance in previous generations and intrafamilial variability. It is commonly associated with a mutation of the CACNA1S (type 1) which encodes the alpha subunit of the voltage gated calcium channel, CaV1.1 (skeletal muscle L-type calcium channel) [9]. Two of three missense mutations in the calcium channel gene account for seventy percent of hypokalemic periodic paralysis cases [10]. About 10–20% is associated with SCN4A (type 2) which codes for the skeletal muscle sodium channel, NaV1.4. NaV1.4 and CaV1.1 are structurally homologous and recent evidence indicates that mutations in both channels are substitution of arginine residues in S4 segments [11]. Mutations in the potassium channel encoded by the KCNE3 gene, expressing an accessory protein that serves to inhibit fast inactivating Kv channel Kv4.3 on chromosome 11q13-q14, have only been reported in one family [12]. Impaired expression of pore-forming Kir6.2 in K_ATP_ channels has been associated with hypokalemic periodic paralysis [13]. Other mutations involving potassium channels such as Kir2.1 (KCNJ2 gene) and Kir2.6 (KCNJ18) have also been found to be linked to decreased excitability of muscles [14, 15].

Complex genetic factors contribute to the pathogenesis of most types of idiopathic epilepsy, but mutations in 11 different genes encoding ion channels account for the majority of epilepsies. Both familial and de novo mutations in neuronal voltage-gated and ligand-gated ion channel subunit genes have been identified in autosomal dominant epilepsies [16]. Mutations in voltage gated sodium, calcium, and potassium channels have recently been associated with idiopathic generalized epilepsy. Voltage gated sodium channels consist of three subunits—a large alpha subunit contains the ion pore and two small accessory beta subunits that modulate kinetics. Mutations in three voltage-gated sodium channel subunit genes are associated with generalized epilepsy. The genes SCN1A, SCN2A coding for the alpha subunit have been linked to specific idiopathic epilepsy syndromes such as generalized epilepsy with febrile seizures plus (GEFS+) and benign familial neonatal-infantile seizures (BFNIS), respectively [17]. Studies have firmly established the relationship between SCN1B, coding for accessory beta subunit, and GEFS+ as well as extending the phenotype to include temporal lobe epilepsy in some families [18]. A de novo heterozygous nonsense mutation in CACNA1A gene encoding the CaV2.1 channel has been reported in a young male patient affected with generalized epilepsy [19]. Other studies show that variation in another calcium channel gene CACNA1H contributes to the pathogenesis of complex epilepsy such as congenital absence epilepsy, but no variants have been described to cause epilepsy on its own [18]. Both voltage gated potassium channels (KCNQ2, KCNQ3) and voltage gated chloride channels (CLCN2) have also been implicated in a range of idiopathic generalized epilepsy syndromes [4].

All forms of familial periodic paralysis share a common final pathway of abnormal depolarization, which inactivates sodium channels and renders the muscle electrically unexcitable. However the specific pathogenesis of calcium, sodium, and potassium channels defects leading to episodic potassium movement into cells resulting in weakness is less clearly understood. Decreased calcium current density, aberrant voltage calcium sensor for excitation-contraction coupling, reduced sarcolemmal ATP-sensitive potassium current, and anomalous sodium current through gated channels have all been proposed as possible mechanisms of hypokalemic periodic paralysis. Although the data on identification of genes underlying idiopathic generalized epilepsy is less clear, similar mechanisms of ion channel dysfunction appear to be the causative factor [20].

We have reported an association between hypokalemic periodic paralysis and idiopathic generalized epilepsy in a family of South Asian descent. Miura et al. first reported the association of generalized epilepsy in a patient with hypokalemic periodic paralysis and cardiac arrhythmias in 1983. They described a “17 year old female with hypokalemic periodic paralysis and syncopal attacks,” which they discovered to be due to idiopathic generalized epilepsy [21]. Two other studies have reported an association of generalized epilepsy and hypokalemic paralysis [22, 23]. We speculate that similar mutations in affecting potassium and calcium channels leading to a defect in gating pore leak may be responsible for a common link between hypokalemic periodic paralysis and generalized idiopathic epilepsy. At present time the precise functional consequences of the CALCNA1 mutations of the CaV1.1 alpha subunit remain unknown. Mutations in the alpha subunits of the CaV1.1 (CALCNA1S) and CaV2.1 (CALCNA1A) have been linked to hypokalemic periodic paralysis and idiopathic generalized epilepsy, respectively. Another possibility is mutations in the Kir6.2, pore-forming K_ATP_ channel subunit, with abundant expression in both muscle and brain. Could a single mutation be responsible for both hypokalemic periodic paralysis and idiopathic generalized epilepsy? Identification of additional families with mutations in pore forming potassium channels and voltage gated calcium channel will be critical to demonstrate the genetic cause of this association. Demonstration of functional consequences in a mammalian assay system could provide additional support for a causal role for these mutations. In addition, because hypokalemic periodic paralysis and epilepsy can result from mutations in several different ion-channel genes, identification of families with disease-causing mutations will be necessary to develop specific therapies for these genetic disorders.

## Figures and Tables

**Table 1:** The onset and clinical presentation of both hypokalemic periodic paralysis and idiopathic generalized epilepsy in the family.

Patient	Age/sex	Age at onset		Serum K+	Frequency of paralysis	EMG	EEG
		HHP	IGE	(During attack)		(During attack)	
I 2	62 F	32	31		1-2 times per year		
II 1	32 M	23	16	2.1 mEq/L	Once a week	CMAP of low amplitude	Bilateral slow wave discharge
II 3	28 M	20	18	2.2 mEq/L	1-2 times a month	Complete electrical silence	Bilateral spike-wave discharge
III 1	11 M	4		1.9 mEq/L	4-5 times a month	CMAP of low amplitude	
III 5	26 M	21	19	1.9 mEq/L	Rare attacks	Complete electrical silence	Bilateral spike- wave discharge

HHP, hypokalemic periodic paralysis; IGE, idiopathic generalized epilepsy; EMG,electromyography; EEG, electroencephalography.
